# Preprocessing Methods for Ambulatory HRV Analysis Based on HRV Distribution, Variability and Characteristics (DVC)

**DOI:** 10.3390/s22051984

**Published:** 2022-03-03

**Authors:** Mouna Benchekroun, Baptiste Chevallier, Dan Istrate, Vincent Zalc, Dominique Lenne

**Affiliations:** 1 Biomechanics and Bioengineering Lab, University of Technology of Compiègne (UMR CNRS 7338), 60200 Compiègne, France; baptiste.chevallier@utc.fr (B.C.); dan.istrate@utc.fr (D.I.); vincent.zalc@utc.fr (V.Z.); 2 Heudiasyc Lab (Heuristics and Diagnosis of Complex Systems), University of Technology of Compiègne (UMR CNRS 7338), 60200 Compiègne, France; dominique.lenne@utc.fr; 3 Core for Tech, 59000 Lille, France

**Keywords:** heart rate variability (HRV), stress monitoring, e-health, wearables, biosensors, ambulatory

## Abstract

Thanks to wearable devices joint with AI algorithms, it is possible to record and analyse physiological parameters such as heart rate variability (HRV) in ambulatory environments. The main downside to such setups is the bad quality of recorded data due to movement, noises, and data losses. These errors may considerably alter HRV analysis and should therefore be addressed beforehand, especially if used for medical diagnosis. One widely used method to handle such problems is interpolation, but this approach does not preserve the time dependence of the signal. In this study, we propose a new method for HRV processing including filtering and iterative data imputation using a Gaussian distribution. The particularity of the method is that many physiological aspects are taken into consideration, such as HRV distribution, RR variability, and normal boundaries, as well as time series characteristics. We study the effect of this method on classification using a random forest classifier (RF) and compare it to other data imputation methods including linear, shape-preserving piecewise cubic Hermite (pchip), and spline interpolation in a case study on stress. Features from reconstructed HRV signals of 67 healthy subjects using all four methods were analysed and separately classified by a random forest algorithm to detect stress against relaxation. The proposed method reached a stable F1 score of 61% even with a high percentage of missing data, whereas other interpolation methods reached approximately 54% F1 score for a low percentage of missing data, and the performance drops to about 44% when the percentage is increased. This suggests that our method gives better results for stress classification, especially on signals with a high percentage of missing data.

## 1. Introduction

Heart rate variability quantifies the fluctuations in the time intervals between successive heart beats (RR intervals). The analysis of HRV can provide insights into autonomic nervous function and information about the sympathetic–parasympathetic balance and cardiovascular health [1]. Thanks to machine learning algorithms and wearable biosensors, HRV is widely used today as an indicator of different physiological states and pathologies such as mental stress [2,3].

HRV data collection is relatively easy, noninvasive, and inexpensive, which makes it valuable and very popular for ambulatory health monitoring [4]. HRV can be extracted from either ECG or PPG sensors that are widely available today.

Whereas HRV analysis requires accurate RR interval (RRI) time series including only pure sinus beats, wearable type ECG and PPG devices readily generate artifacts and important data loss, which cause gaps and abnormal RR intervals. Because HRV features derived from bad quality signals cannot be trusted for a reliable classification, especially if used for medical purposes, HRV signals should be carefully edited for data imputation and miscalculated RRI exclusion beforehand as emphasised by many studies [5,6,7].

Commonly used methods for data imputation in HRV signals include linear, cubic spline, and cubic Hermite interpolation [8]. These methods are efficient for classification with low percentages of missing data. However, they do not perform well with low quality signals.

In this paper, the pipeline for stress detection from ECG and PPG is carefully presented from data collection to stress classification through all signal processing steps. We propose a new HRV processing method including two processes: filtering ectopic RRI, and replacing missing data. In order to test the efficacy of our method, RR time series are degraded and reconstructed. An increasing percentage of data is deleted from the original signal and then handled by different existing methods, such as linear, spline, and pchip interpolation [9] together with our method introduced below. A random forest classifier is then tested on each dataset (from each data imputation method) and classification is compared through performance metrics to compare the impact of these imputation methods on stress classification.

### 1.1. Related Work

Because of the growing interest in HRV for ambulatory health monitoring [10], many studies have been carried out to find methods to tackle diverse errors and important data losses during acquisition, transmission, or data storage. These errors alter the signal and can introduce an important bias in HRV analysis when not addressed beforehand [8].

In addition to data losses, ectopic beats also have an impact on HRV features. Authors in [11] found the presence of only one ectopic beat in a 2 min ECG recording to increase some HRV features by approximately 10%. Ectopic beats also cause erroneously higher values of the standard deviation of the RRI time series  [12]. These errors are not acceptable for a precise HRV analysis potentially used for medical diagnosis.

Ectopic beats are defined as RR intervals shorter than 300 ms (i.e., 200 bpm) or longer than 1300 ms (i.e., 46 bpm). They might be caused by a physiological phenomenon such as premature ventricular contractions (PVC) or premature atrial contractions (PAC) [13,14,15], but most of the time they occur due to a false peak detection on PPG or ECG signals or due to a missed beat.

Studies on the subject suggested different methods for dealing with ectopic beats, including deletion and interpolation. The easiest approach is the deletion procedure. Its main downside however is the signal depletion, as deleted values are not replaced. This approach also increases the abrupt changes in the signal and introduces disruptions in its natural fluctuation [16]. Moreover, resampling, which is essential for analyzing HRV in the frequency domain, may produce outliers if the RRI time series contains missing values [17]. In addition, for signals recorded in ambulatory environments, the deletion approach is not suitable because there is already a high percentage of missing data.

By far the most popular data imputation method for HRV is interpolation, linear and cubic spline, particularly [18,19]. Although interpolation can help roughly preserve or recover recording duration, it does introduce changes and outliers that affect HRV analysis. Interpolating linearly may lead to false decreased variability [20], whereas splines produce outliers due to oscillation of its interpolation function [17]. Authors in [21] found that interpolation also introduces low frequency components (LF) and reduces high-frequency components (HF) power of the signal. Conventional HRV processing generally includes both deletion and interpolation. Non physiological beats are deleted and then replaced by interpolated values.

### 1.2. Paper Contribution

The method we propose goes beyond the mathematical aspect of data imputation to take different physiological constraints into consideration. Three aspects are preserved, including normal limits of RR intervals (300 ms to 1300 ms), HRV distribution, and variability, as the new values are generated using a Gaussian distribution, whose parameters are computed from the data themselves. Finally, the iterative process when filling a gap preserves HRV time dependence and signal properties and guarantees that all inserted RRI are indeed in the physiological boundaries of 300 ms to 1300 ms.

In this paper, we propose a new approach for HRV processing and we measure its impact on stress classification, as classification is the ultimate goal. We compare the effect of different methods for data imputation on stress classification performances, whereas most of the studies published today are limited to comparing original and reconstructed signals in terms of HRV features and relative errors.

Our approach seems suitable for processing HRV signals with a high percentage of missing data such as those recorded in ambulatory environments thanks to wearables. This allows us to take advantage of poor quality data that would otherwise yield unreliable classification results.

## 2. Materials and Methods

### 2.1. Data Collection

As our main purpose is to evaluate the effect of HRV preprocessing on stress classification performances, we decided to test our method using a dataset from our study on mental stress. This study was carried out in The INSEAD-Sorbonne Université Behavioural Lab in Paris and the protocol was approved by their Review Board (IRB: 202077).

The study was performed under laboratory conditions, where the environment is controlled and movement is reduced. Subjects were selected through an inclusion questionnaire in accordance with ethical criteria as well as study constraints. Volunteers who did not meet all the inclusion criteria, such individuals with chronic diseases (hypertension, diabetes, etc.), cardiovascular diseases, or mental disorders (depression, anxiety, etc.) were excluded. Subjects were asked to abstain from alcohol, caffeine/theine, and tobacco for 12 h, 4 h, and 2 h, respectively, before the experiment.

During each session, four types of physiological signals were recorded, using Shimmer Sense sensors, including electrocardiogram (ECG), photoplethysmogram (PPG), electrodermal activity (EDA), and electromyogram (EMG) on the trapezius muscle, but only two (ECG and PPG) are used for HRV assessment in this paper.

Subject were recorded in two different states:

1. Relaxation: Subjects followed guided meditation for 15 min via an audio track with closed eyes, while sitting in a comfortable position, in a dark environment.
2. Stress: Participants perform stressful tasks such as the Stroop color word test, mental arithmetic, and a speed game, all proven to induce mental stress, for about 20 min [22,23]. A red timer and a visible score were used as social threats to increase the stress response. In addition, subjects were not aware that this step was to induce stress. Instead, they were told an IQ score will be computed to compare them to subjects of the same age category. This is perceived as a threat to one’s social esteem or social status, which activates the stress response as supported by the Social Self-Preservation Theory [24,25].

Protocol validation was achieved using both salivary cortisol levels and psychological questionnaires (State and Trait Anxiety Inventory). More details about the experimental protocol can be found at [26].

### 2.2. Signal Prepocessing

**Figure 1:**
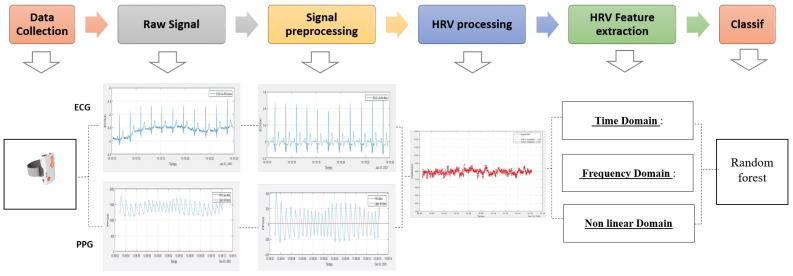
Overview of processing steps from data collection to HRV feature extraction and classification. Each of these steps is detailed in Section 2.

Figure 1 shows an overview of the procedure to identify mental stress based on physiological signals collected from ECG and PPG biosensors.

Although wireless biosensors are designed to capture various biosignals passively and continuously, they also capture a significant amount of unwanted and unknown noises from body and sensor’s movements as well as environmental noise that affect the signal of interest. As such sensors were used in our experiment, the first step is to reduce artifacts by applying various filtering methods.

#### 2.2.1. ECG Processing

A third-order Butterworth bandpass filter [5 Hz–150 Hz], a discrete wavelet transform (DWT) (Db4) with hard thresholding as well as a 50 Hz notch filter were applied to the raw ECG signal to remove both high frequency noise and the power line. Finally, R peak detection was achieved using an optimised Pan–Thompkins algorithm on MATLAB [27].

#### 2.2.2. PPG Processing

PPG signal can be divided into two components, pulsatile (AC) and superimposed (DC). The AC component is provided by the cardiac synchronous variations in blood volume and is used to compute HRV. It is extracted using a band-pass filter. Cut-off frequencies are carefully selected so as not to distort the signal and so that the DC component is no longer dominant. As most of the energy of PPG signal is below 10 Hz, a second-order Butterworth filter [0.5 Hz–10 Hz] was used to remove both high frequency noises (motion artifacts) and the baseline drift [28]. Pulse peak detection was achieved using the Find peak function on MATLAB.

### 2.3. Proposed Method for HRV Processing Based on HRV Distribution, Variability, and Characteristics DVC

HRV signal was extracted from both PPG and ECG by computing the time difference between two successive beats. When signal quality is low, as is the case in ambulatory recordings, additional processing steps need to be undertaken to handle gaps as well as ectopic beats caused by false automatic peak detection on PPG and ECG signals.

Standard HRV processing consists of deleting non physiological RR intervals (RR > 1.3 s or RR < 0.3 s) followed by a mathematical interpolation to replace deleted and missing values (Section 1.1). This approach, however results, in the loss of the time dependence and biased variability [20].

**Figure 2:**
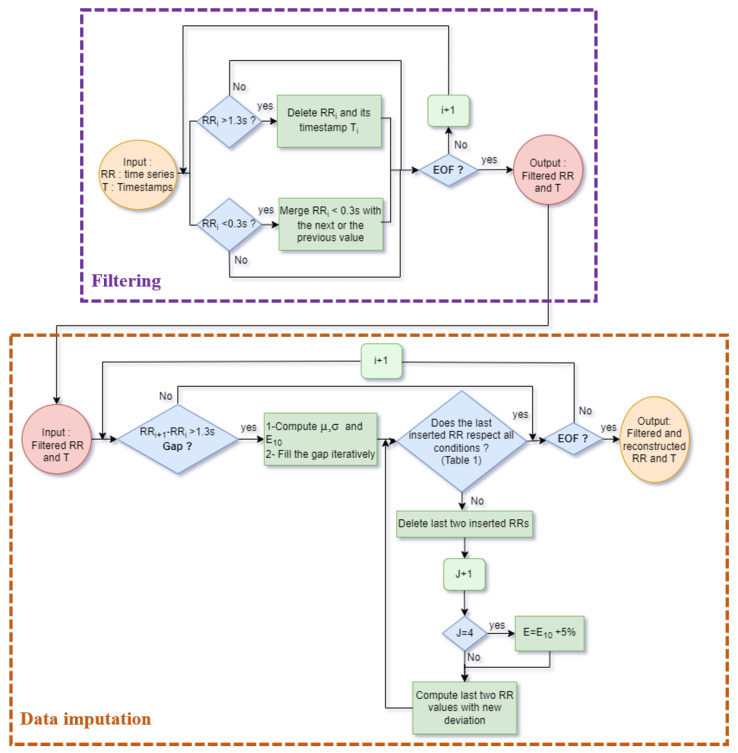
Flowchart for DVC algorithm including filtering and data imputation processes.

In the following section, we suggest a different processing approach for HRV signal imputation including two processes: filtering ectopic RRI and replacing missing data. Figure 2 depicts both processes in a flowchart.

#### 2.3.1. Ectopic Beats Filtering

In our method, *RR* > 1.3 s are deleted, whereas *RR* < 0.3 s are merged with the previous or the next RRI in accordance with three physiological conditions to be observed (Table 1).

The particularity of an HRV signal is the equality between the ordinate of each point and the difference of its abscissa and the previous abscissa value, where the abscissa is RRI timestamp (Equation (10)).

(1) $$\begin{array}{c} RR_{i}=T_{i}-T_{i-1}. \end{array}$$

When an *RR* interval is deleted without proper replacement, this characteristic is lost. Contrary to deletion, this filtering method preserves time dependence and takes into account both past and future in the process when adding the *RR* < 0.3 s to the previous RR interval or the next one.

Our hypothesis here is that small *RR* intervals < 0.3 s are due to an additional peak detected in between two physiological peaks on PPG or ECG signals.

When the *RR* < 0.3 s is removed, either the previous or the next RRI should be modified to preserve the equality in Equation (10).

In the right merge, the new value $RR_{j}\;=\;RR_{i}+RR_{i+1}$ is at $T_{i+1}$ and its predecessor is $RR_{j-1}$ = $RR_{i-1}$. This is called the right merge, as $RR_{i}$ < 0.3 s is added to/merged with the following RR interval (Equation (2)).

**Figure 3:**
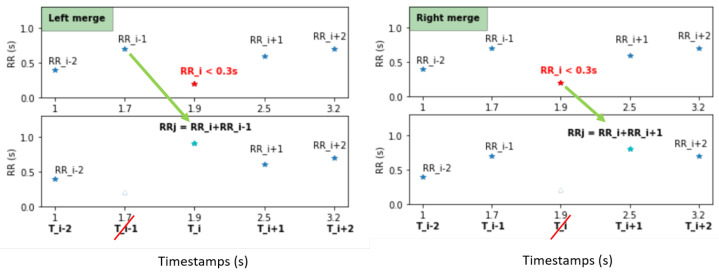
In the right merge, $RR_{i}$ < 0.3 s is added to the next value, and $RR_{i}$ and its timestamp $T_{i}$ are deleted. The new value $RR_{j}=RR_{i}+RR_{i+1}=T_{i+1}-Ti-1$. In the left merge, $RR_{i}$ is added to $RR_{i-1}$ and placed at $T_{i}$, and $RR_{i-1}$ as well as its timestamp $T_{i-1}$ are deleted to respect the equality abscissa-ordinate. The new value $RR_{j}=RR_{i}+RR_{i-1}=T_{i}-Ti-2$. *The subscript i is used to index initial RR intervals and the new value is referred to as $RR_{j}$*.

The exact same process is followed in the left merge, except the new value $RR_{j}\;=\;RR_{i}+RR_{i-1}$ is placed at $T_{i}$ to preserve the time dependence and abscissa and ordinate equality. The predecessor in this case is $RR_{j-1}$ = $RR_{i-2}$ (Equation (3)). Figure 3 depicts this process.

(2)  RRi=Ti−Ti−1, Right Merge+⟹RRj=RRi+RRi+1=Ti+1−Ti−1 RRi+1=Ti+1−Ti, 

(3)  RRi=Ti−Ti−1, Left Merge+⟹RRj=RRi+RRi−1=Ti−Ti−2 RRi−1=Ti−1−Ti−2, 

where $RR_{i}$ is the RR interval at index *i* and $T_{i}$ its corresponding timestamp in time units; $RR_{j}$ is the new value after the merge.

In order to choose which of the merges is suitable, our algorithm goes as follows: the first step is to merge RR < 0.3 s with the following value (right merge in Figure 3) and test the three physiological conditions, detailed in Table 1, on the new value $RR_{j}$ = $RR_{r}$. If the three conditions are not met then the right merge is not possible. A left merge (Figure 3) is tested $RR_{j}$ = $RR_{l}$ with the same conditions. If both the generated $RR_{l}$ and $RR_{r}$ are higher than 1.3 s then the original *RR* < 0.3 s as well as the next value are deleted together with their timestamps.

**Table 1:** Physiological conditions for RRI.

1. 0.3 s < $RR_{j}$ < 1.3 s,
2. Deviation ($E_{r}$) between the new $RR_{j}$ and the following RR interval must be lower than deviation computed over last 10 values
where:
(4) $$E_{r}=\left|\frac{RR_{j+1}-RR_{j}}{RR_{j}}\right|\leq E_{10}\leq 0.4,$$ 3. Deviation ($E_{l}$) between the new $RR_{j}$ and the following RR interval must be lower than deviation computed over last 10 values
where:
(5) $$E_{l}=\left|\frac{RR_{j}-RR_{j-1}}{RR_{j-1}}\right|\leq E_{10}\leq 0.4.$$

If $RR_{j}$ is in physiological boundaries, the deviation between the generated $RR_{j}$ and its predecessor and successor must be lower than the deviation computed over the last 10 RR intervals (Equation (6)). In case the latter is higher than 40%, the maximum deviation is fixed to 40%, which is two times the maximum difference between successive normal RR intervals [29].

(6) $$E_{10}=\frac{1}{10}\sum_{i}^{i+10}\left|\frac{RR_{i}-RR_{i-1}}{RR_{i-1}}\right|\leq 0.4.$$

To make sure our algorithm always converges, conditions two and three (from Table 1) might be dropped if they can not be met as long as the new value 0.3 s < $RR_{j}$ < 1.3 s. In case both $RR_{l}$ < 1.3 s and $RR_{r}$ < 1.3 s but variability is higher than 40% on both sides, then a total error for each merge (right merge and left merge) are computed according to Equation (7) below:

(7) $$E_{tot}=E_{r}+E_{l},$$

for $E_{r}$ and $E_{l}$ greater than 0.4. This means that in case deviations are higher than 40%, we keep the value with the smallest deviation.

The whole filtering procedure is detailed in the pseudo-code below (Algorithm 1).

#### 2.3.2. Data Imputation

Instead of interpolation, missing data are filled by randomly generated RRIs following a Gaussian distribution. Because the heart’s variability depends on several physiological factors, it can be considered as a random signal in the short term, hence the use of a Gaussian distribution. The distribution’s parameters (σ, μ) are computed over the last 10 RRIs before the gap. This allows the generated data to follow the same trend as the previous *RR* intervals while preserving the random aspect contained in physiological data.

This method’s reproducibility is limited as it is based on random values, but the result always follows the data’s distribution.

Thanks to an iterative filling process, the introduced RRI actually corresponds to the time difference between the two peaks (i.e., two successive timestamps), which is not always the case with other standard methods commonly used today.
**Algorithm 1** HRV filtering procedure1:**for** each $RR_{i}$ < 0.3 s **do**2: Compute $RR_{r}=RR_{i}+RR_{i+1}$, $ER_{r}$ (Equation (4)), $ER_{l}$ (Equation (5)) and $Etot_{r}$ (Equation (7))3: 4: **if** $RR_{r}<$ 1.3 s and $ER_{l}\leq E_{10}$ and $ER_{r}\leq E_{10}$ **then**5:  Right merge: Replace $RR_{i+1}$ by $RR_{r}$ and delete $RR_{i}$ and its timestamp6: 7: **else** Compute $RR_{l}=RR_{i}+RR_{i-1}$, and $EL_{r}$ (Equation (4)), $EL_{l}$ (Equation (5)) and $Etot_{l}$ (Equation (7))8:   **if** $RR_{l}<$ 1.3 s and $EL_{l}\leq E_{10}$ and $EL_{r}\leq E_{10}$ **then**9:   Left merge: Replace $RR_{i}$ by $RR_{l}$ and delete $RR_{i-1}$ and its timestamp10: 11:  **else if** $RR_{r}$ > 1.3 s and $RR_{l}$ > 1.3 s **then**12:   Delete both $RR_{i}$ and $RR_{i+1}$13: 14:  **else if** $RR_{r}$ < 1.3 s and $RR_{l}$ > 1.3 s **then**15:   Replace $RR_{i+1}$ by $RR_{r}$ and delete $RR_{i}$ and its timestamp16: 17:  **else if** $RR_{r}$ > 1.3 s and $RR_{l}$ < 1.3 s **then**18:   Replace $RR_{i-1}$ by $RR_{l}$ and delete $RR_{i}$ and its timestamp19: 20:  **else if** Both $RR_{l}$ and $RR_{r}$ < 1.3 s but errors $Etot_{r}$ and $Etot_{l}$ are higher than 0.4 **then**21:   Keep the one with the smaller error22:  **end if**23: **end if**24:**end for**

**Figure 4:**
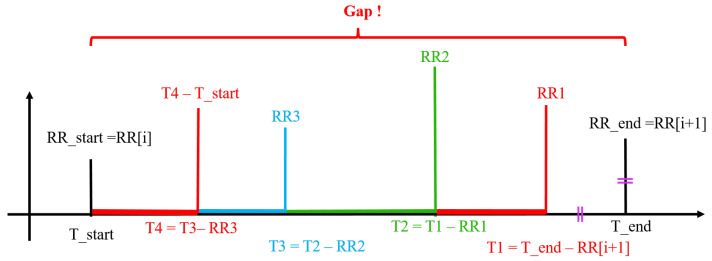
Data imputation using the DVC method. In the first iteration, *T*_1_ is computed and *RR*_1_ is randomly generated. This same process is repeated until $T_{4}-T_{start}$ < 1.3 s and the last *RR* is the time difference between the last two timestamps.

Data imputation is performed iteratively from the end of the gap ($T_{end}$) to its start ($T_{start}$). Once a gap is identified, an RRI respecting physiological conditions enunciated in Table 1 is generated and inserted in the RR time series. The timestamp is then computed as depicted in Figure 4 using the equality property in Equation (10).

Based on Equation (10), the first generated RR interval ($RR_{1}$) should be inserted at:

(8) $$T_{1}=T_{end}-RR_{end}\;\mathrm{so}\;\mathrm{that}\;RR_{end}=T_{end}-T_{1},$$

where $T_{end}$ is the timestamp in the end of the gap and $RR_{end}$ its RR interval (also referred to as R[i+1] in Figure 4); $T_{1}$ is the timestamp right before $T_{end}$ and $RR_{1}$ the RR interval randomly generated and inserted at $T_{1}$ (Figure 4).

The same goes for all other generated RR intervals, such as $RR_{2}$, whose timestamp is computed:

(9) $$T_{2}=T_{1}-RR_{1}\;\mathrm{so}\;\mathrm{that}\;RR_{1}=T_{1}-T_{2}.$$

At each iteration the time difference between the computed timestamp *T* and the timestamp at the start of the gap $T_{start}$ is assisted. Once $T-T_{start}$ < 1.3 s ($T_{4}$ in Figure 4), then the corresponding RR interval is not randomly generated but rather computed as follows:

(10) $$RR_{4}=T_{4}-T_{start}.$$

This last value is then verified to see if it fits all three conditions in Table 1. If it does, then the algorithm moves on to the next gap; if not, the last two values are deleted and regenerated. To make sure the algorithm always converges, after four tries (empirical choice), the deviation is increased by 5%. By doing so, we make sure all RRI are in the [0.3–1.3 s] interval as there is always a solution with two RRIs (0.3 s < *RR* < 1.3 s) in a gap of 1.3 s, although the deviation might be higher than 40%.

### 2.4. HRV Feature Extraction

HRV analysis was performed using the Python Toolbox HRV [30]. Time frequency and non linear domain features are computed from 5 min segments with a 1 min sliding window. The sliding window avoids edge discontinuities and is more suitable for real-time HRV analysis.

#### 2.4.1. Time Domain

Two widely used timed domain features are computed including standard deviation of normal to normal beats (SDNN) (Equation (11)) and root mean square of successive difference between normal heartbeats (RMSSD) (Equation (13)).

Standard deviation of RR intervals (SDRR) is a variation of SDNN that includes abnormal and false beats. SDRR is commonly referred to as SDNN as is the case in this study because ectopic beats eventually introduced during gaps interpolation are not filtered out.

Reflection of both sympathetic nervous system (SNS) and parasympathetic nervous system (PNS) activity can be measured on SDNN, which makes it one of the most useful features of HRV analysis [1]. RMSSD, in contrast, is associated with PNS activation more so than SDNN.

(11) $$\mathrm{SDNN}=\sqrt{\frac{\sum_{i=1}^{N}{\left(RR_{i}-\bar{RR}\right)}^{2}}{N-1}},$$

where

(12) $$\bar{RR}=\frac{1}{N}\sum_{i=1}^{N}\left(RR_{i}\right).$$

(13) $$\mathrm{RMSSD}=\sqrt{\frac{\sum_{i=1}^{N-1}{\left(RR_{i}-RR_{i+1}\right)}^{2}}{N-1}},$$

Another feature computed from successive RR interval differences is the NN50, which is the number of successive intervals differing more than 50 ms or the corresponding percentage PNN50:

(14) $$\mathrm{PNN}50=100\%\times \frac{NN50}{N-1}.$$

#### 2.4.2. Frequency Domain

In this study, frequency domain analysis is performed using both fast Fourier transform (FFT) and discrete wavelet transform (WT) on signals re-sampled at 8 Hz. HRV spectrum is aggregated into three main frequency bands: ultra low frequency (≤0.003 Hz), very low frequency (0.003–0.04 Hz), low frequency (0.04–0.15 Hz), and high frequency (0.15–0.4 Hz) [1], (ULF, VLF, LF, and HF respectively).

As ULF and VLF generally require long periods of recording they are not suitable for real-time analysis and will not be included in this study. Moreover, their physiological correlates are still unknown, which makes them less relevant for e-health applications and for stress detection particularly.

HF and LF, in contrast, can be assessed in shorter time periods (1 and 2 min windows, respectively) [1]. Their correlation with the autonomic nervous system (ANS) and the overall cardiac health has been proven by many studies [31,32] in different contexts, including stress [33].

#### 2.4.3. Non Linear Domain

**Figure 5:**
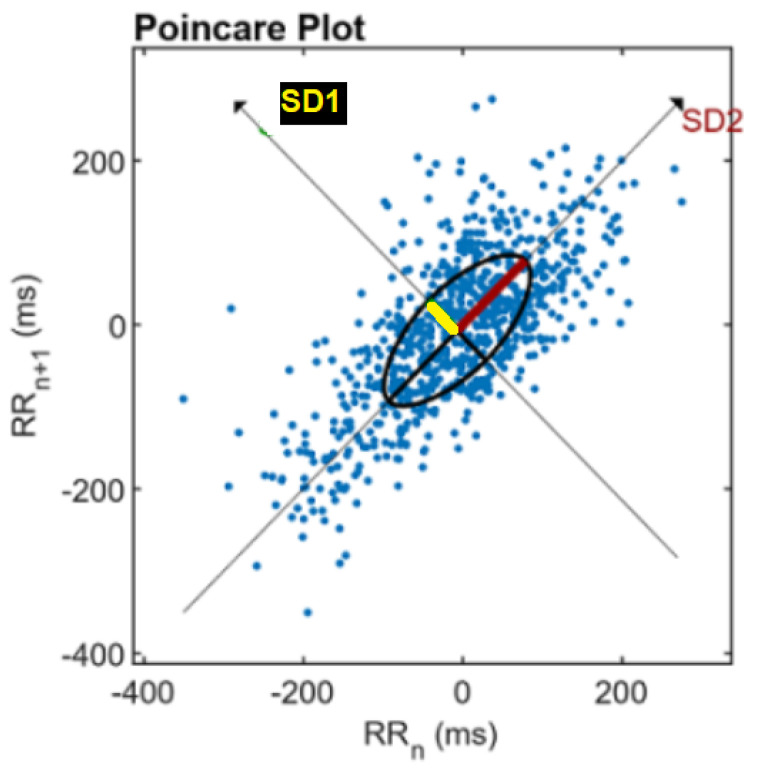
Poincaré plot analysis with the ellipse fitting procedure. SD1 and SD2 are the standard deviations in the directions $x_{1}$ and $x_{2}$. Adapted with permission from [35]. 2016–2021 Kubios Oy.

HRV is regulated by complex mechanisms that sometimes produce non-predictable time series. Therefore studying non-linear features may re-enforce the analysis of such signals. In this study we chose a Poincaré plot, which represents each RR interval against the previous one. It is useful for the visualization of the evolution of a dynamical system in the phase space and for the identification of some hidden patterns [34]. An ellipse can be fitted to the scatter plot of the Poincaré, and two features can be derived: SD1 and SD2, the standard deviations in the directions $x_{1}$ and $x_{2}$, respectively, as can be seen in Figure 5. SD1 measures short-term HRV in milliseconds and correlates with baroreflex sensitivity, which is the change in RR intervals duration per unit change in BP. SD2 measures both short- and long-term HRV in milliseconds [31].

### 2.5. Classification Model

A random forest classifier (RF) is used for stress classification in this study. This model was chosen for many reasons. First, over-fitting can be prevented thanks to pre-pruning techniques by prematurely stopping the growth of the decision tree. Second, the random forest algorithm is stable with high numbers of features. Finally, it is a very popular model among scientists, providing good results with simple hyper-parameters optimization.

The model was implemented using the ‘RandomForest’ package from the sklearn library on Python. We start by randomly splitting the dataset from the original signals into 80% training and 20% validation data with a "stratify" condition on the target column to have approximately the same percentage of samples from each target class. A grid search with a 10-fold cross validation was then performed. The hyper-parameters of the decision tree including the minimum number of samples required to split an internal node (min-samples split) and the minimum number of samples per leaf node (min-samples-leaf) are tuned to early stop the growth of the tree and prevent the model from over-fitting (as part of the pre-pruning technique). The number of decision trees grown based on a bootstrap sample of the observations (n-estimators) and the number of features to consider when looking for the best split (max features) were also tuned in the grid search. The RF model that gave the highest F1 score in the grid search was used on the validation set.

F1 score is chosen to evaluate our model because it combines both precision and recall and is suitable for balanced datasets such as ours.

A feature engineering approach was used to generate new features from the initial set of features detailed in Section 2.3. Each parameter was divided and multiplied by the others. For example, new features include $\frac{\mathrm{RMSSD}}{\mathrm{SDNN}}$, RMSSD × SDNN, $\frac{\mathrm{RMSSD}}{\mathrm{LF}}$, RMSSD × LF … Not all engineered features have a physiological explanation, but the RF classifier is able to choose those that are more relevant to the classification when computing the significance of each attribute before splitting the data. The others will simply not be used by the model.

### 2.6. Validation

In order to test and validate the efficacy of our method against other existing HRV processing approaches, the original signal was degraded and reconstructed using all four methods: DVC, linear, pchip, and spline interpolation. For the last three (linear, pchip, and spline), the number of missing RRI in each gap is estimated based on the mean RR computed over the last 10 values as follows:

(15) $$N=\mathrm{Floor}\mathrm{value}(\frac{\mathrm{Gap}\mathrm{duration}}{\mathrm{Mean}\mathrm{RR}\mathrm{value}}).$$

Once the number of missing values is defined, degraded RR time series are interpolated using the interpolate function from the Python toolbox Pandas, which replaces NaN values using interpolation methods. A dataset with HRV features from each imputation method was generated.

The RF model was trained on the original dataset containing features extracted from raw HRV signals with little missing data and ectopic RR intervals (less than 1% and 2% on average, respectively). It was then tested on features derived from degraded HRV signals processed by our DVC method as well as linear, pchip, and spline interpolation.

For each percentage of deleted data, the validation set is consistent between all the methods to make sure no data from the training set leak into the validation set and to have the same basis of comparison for all four datasets. Methods are compared using F1 scores (16) on the validation set. The f1_score function from sklearn library was used to compute the F1 score.

(16) $$F1=\frac{\mathrm{TP}}{\mathrm{TP}+\frac{1}{2}\left(\mathrm{FP}+\mathrm{FN}\right)}.$$

where

TP = True Positive, HRV windows from stress classified as stress,
FP = False Positive, HRV windows from relaxation classified as stress,
FN = False Negative, HRV windows from stress classified as relaxation.

F1 score value can vary from 0 to 1. The closer the F1 score is to one, the better the classification. This procedure helps identify the best HRV processing method for classification based on real-life, bad quality signals.

## 3. Results and Discussion

In this paper, 68 RR time series in a relaxation state (15 min each) and 67 in a stress state (20 min each) were analysed, for a total of 1510 windows of 5 min.

**Figure 6:**
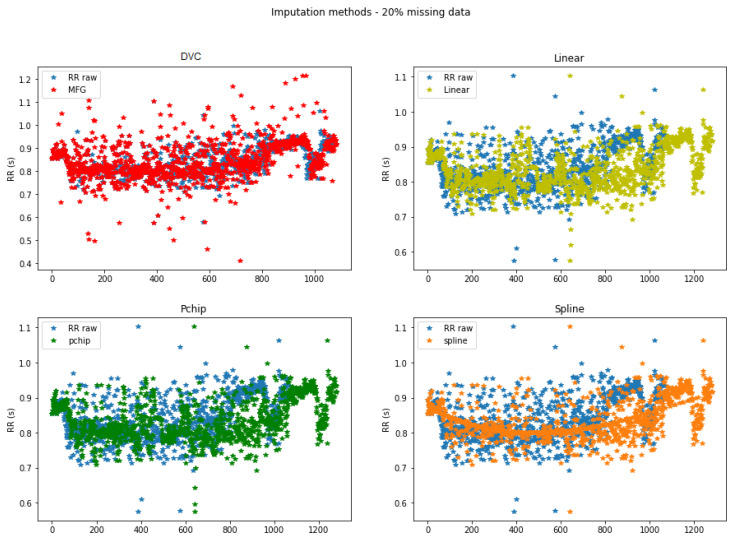
Example of data imputation for 20% deleted data.

An increasing percentage of data (5% to 35%) was randomly deleted and replaced by the proposed DVC method explained in Section 2 as well as the standard methods including linear, pchip, and spline interpolation. Five percent of the data were also replaced by ectopic intervals smaller than 0.3 s. Figure 6 below shows examples of reconstructed RR time series. Four HRV datasets were generated, one for each interpolation method.

As can be seen in Figure 6, there are more RR intervals in the signals reconstructed by linear, pchip, and spline interpolation compared to the original RR time series. This may be because interpolated RR intervals do not necessarily correspond to the time difference between timestamps when using mathematical interpolation. This causes a time lag between the two signals, as more data than actually exist are inserted into each gap. In contrast, the DVC method preserves time series length and time dependence thanks to the iterative process of gap filling.

**Figure 7:**
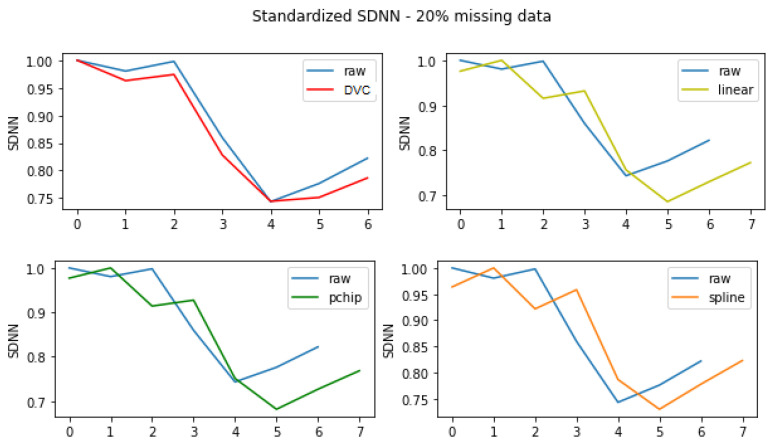
Example of standardized SDNN extracted from the raw and reconstructed signals of 10 min length.

As DVC uses the data’s distribution to generate RRI, the overall data trend is conserved, and time domain features such as PNN50 and SDNN are better preserved, as can be seen in Figure 7.

The grid search on the original dataset yielded the hyper-parameters summarized in Table 2 below.

This model was used to classify arousal states: relaxed vs. stressed using HRV features from raw original data (no degradation) and reached 82% F1 scores on the validation set.

This same RF model was tested on the four other datasets from reconstructed signals using the DVC method, pchip, linear, and spline interpolations. We used the same validation set as the original dataset for all four methods to avoid data contamination.

Table 3 shows F1 scores on validation sets for each method.

The best classification was achieved from HRV signals reconstructed by the DVC method represented in bold in Table 3. This approach turns out to be even more relevant when the percentage of missing data is high. With interpolation, F1 scores quickly drop below 50% when the percentage of missing data exceeds 10%, whereas DVC maintains a 60% F1 score even with 35% of missing data. As can be seen from Table 3, linear and pchip interpolation lose up to 10% F1 scores, whereas DVC keeps steadier performance.

A summary for advantages and disadvantages of each data imputation method used in this paper is presented in Table 4.

## 4. Conclusions

In this work, we propose a new method for processing low quality HRV signals recorded in ambulatory environments. The particularity of our approach is the physiological constraints and characteristics of the HRV signal, which are taken into account in the process of filtering and data imputation. Physiological conditions are observed in the process, such as signal variability and distribution, the heart rate boundaries, and HRV’s characteristics in terms of abscissa and ordinate equality.

The impact on classification of our method is compared to existing interpolations through F1 scores, as the end goal is to suggest an HRV processing approach that gives the best classification results. Higher and steadier F1 scores of approximately 61% were reached using our method compared to 44% (more than 20% missing data) to 54% (5–15% missing data) for other interpolation methods. This proves its efficacy in classification in comparison to other interpolation approaches (linear, spline, and pchip).

In future work, the algorithm presented in this paper can be optimized using more advanced programming methods such as fuzzy logic. Classification using low quality data might also be improved thanks to other classifier such as XGBoost or CatBoost algorithms.

## Figures and Tables

**Table 2:** F1 scores for each data imputation method.

RF Hyper-Parameters
criterion = ’entropy’, max_features = 0.6, min_samples_split = 3, n_estimators = 500

**Table 3:** F1 scores for each data imputation method.

F1 Scores					
**% <0.3 s**	**% Missing**	**DVC**	**Pchip**	**Linear**	**Spline**
5%	5%	**0.63**	0.54	0.53	0.56
5%	10%	**0.62**	0.52	0.51	0.54
5%	15%	**0.61**	0.48	0.47	0.55
5%	20%	**0.61**	0.45	0.45	0.55
5%	25%	**0.61**	0.44	0.43	0.55
5%	30%	**0.61**	0.44	0.43	0.55
5%	35%	**0.61**	0.44	0.43	0.55

**Table 4:** Summary table for advantages and disadvantages of data imputation methods.

Method	Advantages	Disadvantages
Linear	- Assumes less than the other methods - Simple and efficient for good quality signals	- Less effective for signals with lots of missing data - Loss of time dependency
Pchip	- Preserves the linear trend and the slightly non linear contributions in the RR time-series [32]	- Less effective for signals with lots of missing data - Loss of time dependency
Spline	- Can capture abrupt variations when data quality is good	- Introduces outliers due to oscillation of the interpolation function [9] - Less effective for signals with lots of missing data - Loss of time dependency
DVC	- Adaptive to data distribution and variability - No ectopic values in the processed signal - Preserves signal’s time dependency - Effective for low quality signals	- Computationally expensive - Algorithm could be optimised

## Data Availability

The data presented in this study will be made openly available shortly. In the mean time, it is available on request from the corresponding author.
